# Antimicrobial Effect of Silver Nanoparticles Capped with *Killer* Yeast-Derived Protein Fractions

**DOI:** 10.3390/nano16150918

**Published:** 2026-07-26

**Authors:** Carlos Molina-Vera, Verónica Morales-Tlalpan, Juan Campos-Guillén, Jorge Luis Chávez-Servín, Carlos Saldaña

**Affiliations:** 1 Laboratorio de Biofísica de Membranas y Nanotecnología, Universidad Autónoma de Querétaro, Av. De las Ciencias S/N, Juriquilla, Querétaro 76010, Mexico; cal.move12@gmail.com (C.M.-V.); veronica.morales@uaq.mx (V.M.-T.); 2 Laboratorio de Visualización Científica Avanzada, Universidad Autónoma de Querétaro, Querétaro 7601, Mexico; 3 Facultad de Química, Universidad Autónoma de Querétaro, Santiago de Querétaro, Cerro de las Campanas S/N, Querétaro 76010, Mexico; juan.campos@uaq.mx; 4 Facultad de Ciencias Naturales, Universidad Autónoma de Querétaro, Av. De las Ciencias S/N, Juriquilla, Querétaro 76010, Mexico; jorge.chavez@uaq.mx

**Keywords:** green synthesis, bio-assisted synthesis, *killer* toxin, AgNPs, antimicrobials, protein corona, *Saccharomyces cerevisiae*

## Abstract

Recently, novel nanobiotechnological approaches have been developed to reduce the impact of pollutants during the synthesis of nanoparticles (NPs). To do so, green synthesis methods have now become widely adopted, using biomolecules to produce high-quality, stable nanoparticles. In this work, we employed the *K*1 toxin produced by *S*. *cerevisiae* as a reducing and capping agent for silver nanoparticles. The synthesis of Ag-*K*1 NPs was carried out using a concentrated protein fraction from the culture medium of *S. cerevisiae* 42300 containing the secreted *K*1 toxin. The obtained nanoparticles were characterized using UV–Vis spectroscopy, STEM, EDS, and FTIR, and the antimicrobial efficacy was determined against *S. cerevisiae*, *P. aeruginosa*, and *B. subtilis*. The synthesized NPs showed high antimicrobial efficacy, killing all tested strains; additionally, dose–response modeling suggested differential activity among the nanoparticles. This work reports, for the first time, the bio-assisted synthesis and antimicrobial characterization of silver nanoparticles associated with K1 killer toxin-containing protein fractions.

## 1. Introduction

Nanotechnology has become a major driver of technological advances across multiple disciplines. One of the most promising branches in the field is nanobiotechnology, which integrates nanomaterials with biological systems to create compounds and tools to understand biological phenomena [1]. The combination of environmentally friendly synthetic approaches with highly efficient nanoparticle synthesis offers effective alternatives with biological uses, while reducing the impact of pollutants during the synthesis [2,3]. Accordingly, considerable efforts have been devoted to designing nanocomposites that combine biosafety with high biological performance. Here, we developed a silver nanoparticle-based system. Silver (Ag) is commonly used in food safety and biomedical applications [4]. To date, the application of the *K*1 *killer* toxin of *S. cerevisiae* as a capping agent for NPs has not yet been explored, opening a new avenue for research in this field.

The *killer* phenotype of *Saccharomyces cerevisiae* has been extensively investigated because of its capacity to mediate potent antimicrobial activity by its secreted *killer* toxins. Nowadays, it is known that the origin of this phenotype is the secretion of a toxic peptide coded in the virus M1, a satellite of the Totivirus L-A [5,6]. The M1 virus contains a single ORF (Open Reading Frame) of 964 bp, which encodes a preprotoxin (pptox) of four domains: δ, α, γ, and β. During the maturation of the peptide, the subunit γ is glycosylated and both δ and γ are removed in the ER (endoplasmic reticulum) by the peptidases Kex1p and Kex2p [7]. The final product is a mature *K*1 *killer* toxin, a 36 kDa heterodimer α-β stabilized by a disulfide bond [8,9,10]. Previous studies have demonstrated that the lethal activity of *K*1 on sensitive cells (non-producers of *killer* toxins) is related to the α subunit, acting as an ionophore or altering the stability of the potassium channel Tok1p [11,12,13,14,15]. Meanwhile, it has been proven that the principal receptor of this toxin is the β-1-6-D-glucan of the cell wall and the receptor Kre1p, a cell-surface O-glycoprotein [16,17]. Beyond their antimicrobial properties, yeasts have also emerged as efficient biological platforms for the green synthesis of metallic nanoparticles. To date, *killer* strains have not been used to produce any nanoparticles; however, other yeasts have previously been used to produce NPs from several precursors like Ag, Au, and Fe [18,19,20,21,22,23]. Despite these advances, no previous study has explored the use of *killer* yeast strains or *killer* toxin-containing protein fractions for the bio-assisted synthesis or functionalization of silver nanoparticles, representing an important knowledge gap in the development of biologically functionalized antimicrobial nanomaterials. In this study, we investigated the bio-assisted synthesis and surface conjugation of silver nanoparticles using *killer* toxin-containing protein fractions obtained from *S. cerevisiae*. The resulting nanomaterials were physiochemically characterized and evaluated for their antimicrobial activity against bacterial and yeast models. We further analyzed their inhibitory potency by MIC determination and dose–response modeling to elucidate the contribution of the protein corona to their biological activity.

It is important to emphasize that Kcpf represents a complex extracellular biomolecular fraction containing the *K*1 killer toxin along with other secreted proteins and biomolecules naturally present in the *S. cerevisiae* culture supernatant. Accordingly, the biological and antimicrobial properties observed throughout this study are discussed as a collective effect of the overall Kcpf-derived biomolecular corona, where the *K*1 toxin acts as one functionally relevant component within this coating.

## 2. Materials and Methods

### 2.1. Reagents

Silver nitrate (AgNO_3_, ACS grade; Karal S.A. de C.V., León, Mexico) used for nanoparticle synthesis was kindly provided by Dr. Pedro Salas-Castillo (UNAM). Polyethylene glycol 3350 (PEG 3350, ACS grade; Sigma-Aldrich, St. Louis, MO, USA), D-(+)-glucose (ACS grade; Sigma-Aldrich), and ethanol (Sigma-Aldrich) were used as received; for culture media, peptone (Merck KGaA, Darmstadt, Germany), yeast extract (BD DIFCO, México City, Mexico), KH_2_PO_4_ A.C.S. (Macron Fine Chemicals, Avantor Performance Materials, S.A. de C.V. Xalostoc, Edo. De Méx. Mexico), citric acid A.C.S. (Macron Fine Chemicals), agar (BD DIFCO, México City, México) were used; and for bacterial culture, LB broth (Sigma-Aldrich). For PBS buffer, NaCl (Reasol, Mexico), KCl (Macron Fine Chemicals), Na_2_HPO_4_ (J.T. Baker, Mallinckrodt Baker S.A. de C.V. Xalostoc, Edo. De Méx. Mexico), and KH_2_PO4 (J.T. Baker) were used. All reagents were prepared with triple-distilled water with no further purification; meanwhile, the culture media were sterilized at 121 °C for 20 min in an autoclave (AV-3070, Prendo. SEVMÉXICO).

### 2.2. Strains and Culture Media

The *S*. *cerevisiae* strain 42300 (*killer*; MAT α Ade2/+ Thr1/+ sKi2-1/+ [KIL-K1]) (ATCC 1382) and a sensitive yeast strain 5 × 47 (MATa/α his1/+ trp1/+ ura3/+ [K10 K20 K280 Klus0]) (ATCC 38527) were maintained on YPD broth (1% yeast extract, 2% peptone, 2% glucose, 0.1 M KH_2_PO4, pH 4.7 adjusted with citric acid) at 30 °C with continuous shaking for 24 h. *B. subtilis* strain PY79 (kindly provided by Dr. Juan Campos Guillén, FQ, UAQ) and *P. aeruginosa* strain 575 (resistant to imipenem and meropenem; donated by Dr. José Antonio Cervantes Chávez, FCN, UAQ) were maintained on LB broth at 37 °C with continuous shaking for 24 h.

### 2.3. Killer Toxin Induction and Concentration

*K*1 secretion was induced by adding 0.1 g of sensitive yeast per gram of *killer* yeast to a 50 mL fresh YPD broth (pH 4.7). Cultures were incubated at 30 °C, with continuous shaking for 24 h. The culture was then processed with a modified protocol previously described [24]. The culture was centrifuged at 5000 rpm for 10 min, and the supernatant was filtered using a 0.22 µm filter (Milipore) and concentrated with a 10 kDa column (Briscale UF-15, Cobetter, China) [25]. The resulting *killer*-concentrated protein fraction (Kcpf) was stored at 4 °C until further use. The total protein concentration of the resulting *killer*-concentrated protein fraction (Kcpf) was determined using the bicinchoninic acid (BCA) protein assay kit (Thermo Scientific) with bovine serum albumin (BSA) as standard. The harvested Kcpf stock solution (yield: 2 ± 0.5 mg/mL total protein) was diluted in PBS (pH 7.4) to a standardized working concentration of 0.5 mg/mL before nanoparticle synthesis and conjugation procedures.

### 2.4. Synthesis of Ag Nanoparticles

#### 2.4.1. Synthesis of AgNPs with Glucose and PEG

Ag-GluPEG nanoparticles were synthesized using a modified glucose reduction method. In a typical reaction, 8.5 mL of triple-distilled water was heated to boiling under continuous stirring. Then, 1.0 mL of a 20 mM AgNO_3_ stock solution was added to reach a final concentration of 2 mM. After 30 min of heating and stirring, 0.5 mL of a 20% (*w*/*v*) PEG 3350 solution (1% *w*/*v* final concentration) and D-(+)-glucose (from a stock solution to achieve final concentrations ranging from 0.5 to 300 mM; 300 mM for optimal formulation) were added, adjusting the final reaction volume to 10 mL with triple-distilled water. The mixture was maintained at boiling temperature under continuous stirring for an additional 30 min until a characteristic brownish-yellow color developed. The suspension was allowed to cool to room temperature in the dark overnight. The resulting nanoparticles were recovered by centrifugation at 13,000 rpm for 20 min, washed twice with absolute ethanol, twice with sterile triple-distilled water, and finally resuspended in PBS (pH 7.4). The Ag-GluPEG stock suspension was stored at 4 °C in the dark until further use.

#### 2.4.2. Conjugation of AgNPs with Kcpf

Freshly synthesized Ag-GluPEG NPs were conjugated with Kcpf obtained from the previous culture to generate the Ag-Kcap preparation. The conjugation was carried out by passive adsorption, mixing 1 volume of freshly prepared Ag-GluPEG NPs with 1 volume of Kcpf (typically 2 ± 0.5 mg/mL) in a clean vial. This mixture was incubated at room temperature under indirect light for 72 h until the suspension changed from light yellow to golden brown. The conjugated NPs were recovered by centrifugation at 13,000 rpm for 20 min, and washed as previously. The NPs obtained were then stored at 4 °C until use.

#### 2.4.3. Bio-Assisted Synthesis of Ag Nanoparticles with Kcpf

Bio-assisted Ag-Kbs nanoparticles were synthesized using the concentrated protein fraction (Kcpf) as both reducing and stabilizing agent. Briefly, 1.0 mL of a 20 mM AgNO_3_ stock solution (final concentration 2 mM) was mixed with 9.0 mL of freshly harvested Kcpf (2 ± 0.5 mg/mL) to yield a final volume of 10 mL. The reaction mixture was incubated at room temperature (25 °C) under indirect natural light with continuous gentle agitation for 72 h until the color shifted from light golden to brownish amber, indicating nanoparticle formation. The resulting Ag-Kbs nanoparticles were collected by centrifugation at 13,000 rpm for 20 min, washed twice with absolute ethanol and twice with sterile triple-distilled water to eliminate unbound organic residues, resuspended in PBS (pH 7.4), and stored at 4 °C in the dark until use.

### 2.5. Characterization of Ag NPs

Nanoparticle characterization was performed by UV–Vis spectroscopy using the VarioSkan ^®^ Flash (Thermo Scientific, Waltham, MA, USA) operating in spectrophotometric mode at the Proteogenomic Unit, Instituto de Neurobiología, Juriquilla, UNAM, México. The absorbance spectrum was recorded from 200 to 600 nm with a resolution of 2 nm. Several sample dilutions were prepared to obtain optimal absorbance signals within the linear detection range of the instrument. The particle-size distribution, elemental composition, and morphology were measured by High-Resolution Scanning Transmission Electron Microscopy (HR-STEM) and Energy Dispersive X-ray Spectroscopy (EDS) (Hitachi SU8230, Tokyo, Japan, Microscopy unit, Centro de Física Aplicada y Tecnología Avanzada, UNAM, Mexico). The functional groups present on the AgNPs were detected by Fourier-transform infrared (FTIR) spectroscopy over the spectral range of 600 to 4000 cm^−1^ using a Perkin-Elmer Spectrum Two spectrometer (Waltham, MA, USA, CFATA Juriquilla, UNAM, Mexico). All analyses were performed in triplicate; the UV–Vis spectral analysis was graphed using GraphPad Prism 10 (GraphPad Software, San Diego, CA, USA), the FTIR spectra were analyzed in Origin, and the TIFF-converted images were analyzed in ImageJ software (NIH, New York, NY, USA).

### 2.6. Antimicrobial Activity

To determine the effectiveness of the AgNPs, agar well diffusion assays were performed using *S*. *cerevisiae* 5 × 47, *S*. *cerevisiae* 42300, *P*. *aeruginosa* strain 757, and *B. subtilis* strain PY79. Bacterial strains were cultured in LB broth for 24 h, and the inoculant was adjusted to an optical density of OD600 = 0.5 before antimicrobial testing. Antimicrobial activity was evaluated using the agar well diffusion method by measuring the inhibition zones produced by each nanoparticle preparation. The wells were formed by drilling the agar with a sterile 200 µL pipette tip. All microorganisms were inoculated on YPD agar, and 50 µL of each nanoparticle preparation were tested. After 24 h, all the plates were photographed to measure inhibition zones. The inhibition area was calculated by subtracting the area of the agar well from the total inhibition halo area. The inhibition halos were analyzed using GraphPad Prism 10 using one-way ANOVA.

### 2.7. MIC Determination and IC_50_ Modeling

Prior to microdilution assays, nanoparticle stock suspensions were normalized based on total dry mass (mg/mL) following extensive washing steps (centrifugation at 13,000 rpm for 20 min, twice with absolute ethanol and twice with sterile triple-distilled water) to remove unreacted precursors and unbound organic biomolecules. This standardization ensured that initial test concentrations reflected purified nanoparticle nanocomposites. Minimum inhibitory concentration (MIC) values were determined by a two-fold serial microdilution assay performed in sterile 96-well plates as previously described [25]. Briefly, 300 μL of each nanoparticle preparation at the highest concentration was added to the first well of the plate. Two-fold serial dilutions were prepared by transferring 150 μL into 150 μL of fresh YPD medium until the final dilution was reached. After the last transfer, 150 μL was discarded from the final well to maintain equal volumes throughout the plate. Each well was inoculated with 5 µL of microbial inoculum at OD600 = 0.08. The plate was then incubated overnight at 37 °C. MIC values were defined as the lowest nanoparticle concentration at which no visible microbial growth was observed. Additionally, the absorbance at 600 nm was recorded to quantify microbial growth at each nanoparticle concentration.

Absorbance values obtained from the MIC assays were normalized and fitted using a nonlinear dose–response model. Heatmaps were generated in BioRender, whereas dose–response curves and IC_50_ values were calculated using GraphPad Prism 10 (GraphPad Software, San Diego, CA, USA).

## 3. Results and Discussion

### 3.1. UV–Visible and Morphological Characterization of AgNPs

**Figure 1:**
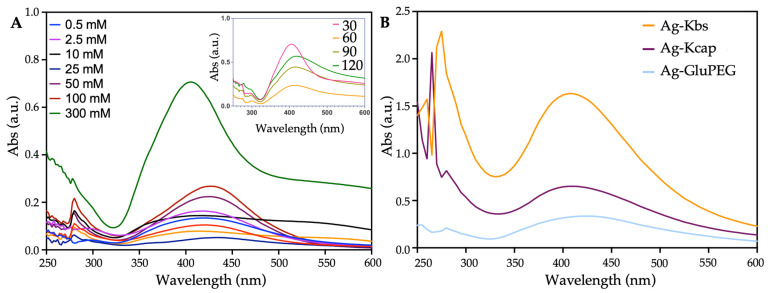
UV–Vis absorption spectra of AgNPs. (**A**) Optimization of Ag-GluPEG synthesis using a D-(+)-glucose concentration gradient (0.5–300 mM) with 2 mM AgNO_3_; inset shows the UV–Vis spectral changes at different reaction times (30, 60, 90, and 120 min). (**B**) UV–Vis spectra of biosynthesized (Ag-Kbs) and passively conjugated (Ag-Kcap) silver nanoparticles with *killer*-derived concentrated protein fractions (Kcpf) compared to Ag-GluPEG. All reaction mixtures contained a final precursor concentration of 2 mM AgNO_3_. Absorbance values are expressed in arbitrary units (a.u.).

The synthesized AgNPs exhibited a characteristic surface plasmon resonance (SPR) band in the UV–Vis spectra (Figure 1) between 380 and 400 nm, confirming the successful formation of silver nanoparticles. Furthermore, the concentration of the reducing agent markedly influenced the SPR profile and, consequently, the nanoparticle synthesis process. Several studies have reported the use of glucose as both a reducing and stabilizing agent during silver nanoparticle synthesis [26,27,28]. In our study, increasing the glucose concentration enhanced the intensity of the SPR band. As shown in Figure 1A, the highest absorbance was obtained using 300 mM glucose and 2 mM AgNO_3_. Interestingly, the signal of those reactions tends to oscillate over time, and when the reactions were carried out for 30 min, they show a stronger SPR signal, reaching a single, well-defined peak at 390–400 nm. However, when the reactions were carried out for 60 min, the SPR showed a lower peak and increased again at 90 and 120 min. Notably, the maximum absorbance at 120 min did not reach the initial peak observed at 30 min (Figure 1A). These results confirm the presence of a typical AgNP signal commonly reported around 400 nm on yeast-assisted synthesis [19,29,30,31]. By contrast, when the NPs were biosynthesized with the Kcpf, the SPR of the AgNPs was significantly increased, and a secondary peak appeared at 280 nm (Figure 1B). This can be attributed to the absorbance of aromatic amino acids such as tryptophan and tyrosine, which mainly contribute to the absorbance spectra of protein samples [32]. Previous reports have demonstrated the usage of UV–Vis spectroscopy for the analysis of complex protein formulations, highlighting signals related to the aromatic amino acids in the region between 260 and 300 nm [33,34,35]. In our system, the yeast produces and secretes *killer* toxin, which is the main protein, into the media [24], contributing to the absorbance in the aromatic region of the synthesized nanoparticles. Remarkably, the capped nanoparticles showed an increased signal around 270 nm and an increase in the 400 nm signal compared to the non-capped nanoparticles. This behavior may be related to the protein decoration and an increase in the NP concentration.

**Figure 2:**
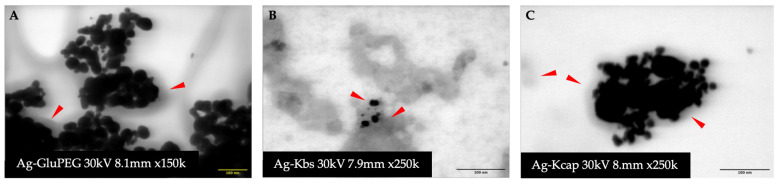
HR-STEM images of AgNPs. (**A**) AgNPs synthesized with glucose and PEG showed a clear coating on the surface. (**B**) Meanwhile, those that were biosynthesized had low electrodense cumulates and NPs of different sizes. (**C**) NPs conjugated with Kcpf conserve the coating and carry low electrodense protein clusters. Scale bars represent 100 nm.

The morphology of the synthesized nanoparticles differed markedly among the different preparations. AgNPs synthesized using glucose and PEG exhibited the most homogeneous particle-size distribution, with an average diameter of 18.4 ± 6.3 nm. In comparison, Ag-Kbs and Ag-Kcap displayed larger average particle sizes of 20.7 ± 5.6 nm and 25.0 ± 16.6 nm, respectively. According to already reported evidence, the use of PEG as a capping agent results in the formation of a uniform coating that can be visualized by high-resolution transmission electron microscopy (HR-TEM) [36,37,38,39]. Consistent with these reports, a well-defined surface coating was observed surrounding the glucose/PEG-synthesized AgNPs (Figure 2A). As expected, the same surface coating was also observed in Ag-Kcap nanoparticles after conjugation with Kcpf. In both preparations, the PEG coating exhibited an average thickness of 29.9 ± 7.4 nm, which is consistent with previous reports for nanoparticles coated with PEG 2000 [37,39] and PEG 4000 [36]. These observations support the reported relationship between PEG molecular weight and coating thickness [40]. Ag-Kbs nanoparticles exhibited a narrow particle-size distribution, which may be attributed to the proteins present during the synthesis reaction. These proteins likely acted as stabilizing agents, promoting a more uniform nanoparticle population. As shown in Figure 2B, the nanoparticles are surrounded by a low electron-dense matrix, which is likely associated with protein aggregates derived from the killer protein fraction. A similar feature was observed in Ag-Kcap nanoparticles, where these protein aggregates were present in addition to the PEG coating (Figure 2C).

These morpho-structural variations play a pivotal role in governing biological activity. The uniform PEG shell in Ag-GluPEG provides steric stability while allowing rapid release of silver ions (Ag^+^), resulting in lower IC_50_ values. In contrast, the denser biological matrix surrounding Ag-Kbs and Ag-Kcap acts as a dynamic diffusion barrier that slows Ag^+^ release. However, this organic layer offers a distinct biological advantage by introducing active targeting moieties from the protein corona that interact directly with microbial cell-wall receptors.

### 3.2. Elemental and Functional Characterization of AgNPs

The elemental composition of the AgNPs was determined by energy-dispersive X-ray spectroscopy (EDS) coupled to SEM. The analysis revealed differences between the biosynthesized and conjugated NPs. In all samples, a characteristic silver signal was detected at approximately 3 keV (kilo-electron volts), confirming the presence of AgNPs on all the samples [41,42]. However, the conjugated AgNPs exhibited additional nitrogen signals, which may be associated with nitrogen-containing organic components from the coating layer or, alternatively, with residual precursor-derived species. In contrast, nitrogen signals were not detected in biosynthesized AgNPs, suggesting an efficient conversion of the silver precursor during the biosynthetic process (AgNO_3_). The analysis also revealed carbon signals, which are commonly associated with the carbon-coated support grid used for SEM-EDS measurements. Additionally, signals corresponding to Na, Mg, Si, K, P, S, and Cl were detected, with marked differences between biosynthesized and conjugated AgNPs (Figure S1). Trace elements such as Mg and Si have previously been reported in EDS analyses of nanomaterials [19]. The presence of Na and other nonmetal elements may be related to residual components from the yeast culture system, including extracellular biomolecules and inorganic ions derived from the growth medium. These differences may reflect the influence of the biological synthesis process and the chemical environment provided by yeast-derived components [43].

**Figure 3:**
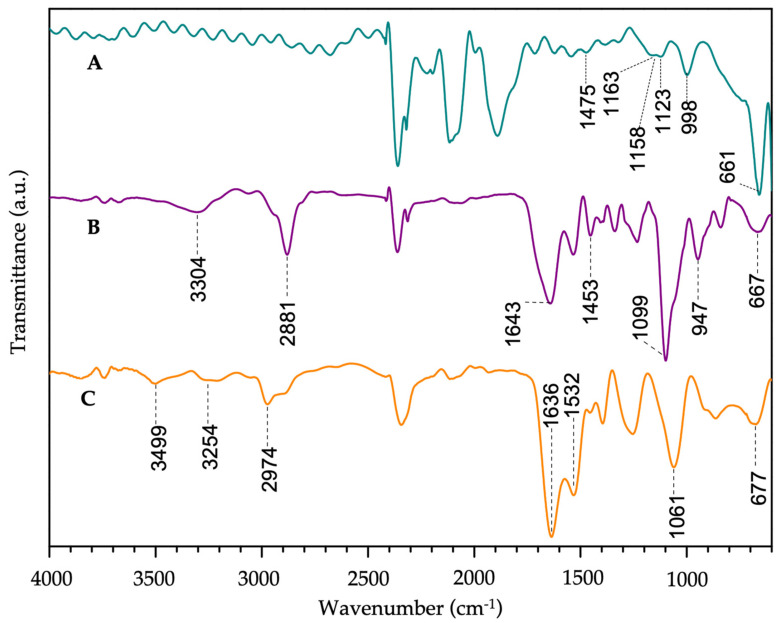
FTIR spectra of AgNPs. (**A**) AgNPs synthesized with glucose and PEG, (**B**) AgNPs capped with Kcpf, and (**C**) AgNPs biosynthesized with Kcpf. Signal intensity is represented in arbitrary units (a.u.).

To identify functional groups associated with the organic coating of AgNPs, Fourier-transform infrared spectroscopy (FTIR) analysis was performed (Figure 3). The spectra obtained for glucose- and PEG-functionalized AgNPs showed characteristic bands associated with PEG and carbohydrate-derived molecules. The band observed at 1475 cm^−1^ was assigned to asymmetric C–H bending vibrations, whereas the signals detected at 1123, 1158, and 1163 cm^−1^ were consistent with C–O–C stretching vibrations from PEG chains [44]. Additionally, a signal at 998 cm^−1^ was associated with C–O vibrations from carbohydrate structures [45], while the band detected at 661 cm^−1^ was attributed to Ag–nonmetal interactions. Previous studies describing AgNPs synthesized using glucose and PEG 3350 reported characteristic glucose-associated signals at 3246 cm^−1^ (O–H), 2901 cm^−1^ (C–H), 998 cm^−1^ (C–O–H/C–O–C), and bands ranging from 1145 to 554 cm^−1^ corresponding to C–O and C–C vibrations. PEG 3350-associated signals were reported at 3441 cm^−1^ (O–H), 2878 cm^−1^ (C–H), 1464 cm^−1^ (C–H bending), 1279 cm^−1^, and 1094 cm^−1^ (O–H and C–O–C vibrations) [46]. In agreement with these previous reports, our spectra showed comparable absorption bands with minor shifts, supporting the presence of PEG and glucose-derived residues associated with the nanoparticle surface. These findings suggest the formation of an organic coating layer that may contribute to nanoparticle stabilization.

For bio-assisted AgNPs obtained using Kcpf, the FTIR spectrum showed characteristic absorption bands associated with protein- and carbohydrate-derived functional groups. Specifically, the broad band centered at 3499–3254 cm^−1^ corresponds to primary O–H and N–H stretching vibrations from protein peptide backbones and carbohydrate hydroxyl groups [47,48,49]. The prominent absorption bands at 1636 cm^−1^ and 1532 cm^−1^ are characteristic of amide I (C=O stretching) and amide II (N–H bending combined with C–N stretching), respectively, confirming the active participation of extracellular proteins in stabilizing the nanoparticle surface [50]. Furthermore, signals observed in the 1200–1400 cm^−1^ region correspond to amide III vibrations, whereas the peak at 1061 cm^−1^ is attributed to C–O stretching vibrations from yeast wall-derived polysaccharides and glycans [51]. Furthermore, the band observed at 677 cm^−1^ may be related to Ag–nonmetal interactions [52].

For Kcpf-conjugated AgNPs, the FTIR spectrum showed a hybrid profile combining features of both Ag-GluPEG and Ag-Kbs preparations, which indicated the formation of a complex coating involving proteins, saccharides, and PEG. The bands detected in this formulation were similar to those observed for biosynthesized nanoparticles, including protein-associated bands at 3304 (O–H, N–H), 2881 (C–H), 1643 (amide I), and 1534 cm^−1^ (amide II), confirming the presence of the Kcpf on the outer layer of the NPs [53]. As expected, additional signals at 1453 and 1099 cm^−1^ (C–H bending of PEG chains) and 1099 cm^−1^ (C–O–C stretching) were detected, which are consistent with PEG-associated vibrations. The carbohydrate-associated region, represented by a signal at 947 cm^−1^, was also observed. Moreover, the Ag–nonmetal interaction band was detected at approximately 667 cm^−1^, similar to that observed in glucose/PEG-functionalized AgNPs. The simultaneous presence of PEG, carbohydrate, and protein-associated signals suggests the formation of a mixed organic surface layer composed of PEG and Kcpf-derived biomolecules.

### 3.3. Antimicrobial Effect of AgNPs

The antimicrobial effect of the synthesized nanoparticles was analyzed by a well diffusion test on agar plates. The images were analyzed for inhibition zone determination by subtracting the area of the well from the total inhibition halo.

It has been previously reported that yeast-mediated AgNPs had significant antibacterial activity [20,21,30,54,55]. However, to date, there is no evidence reported of the usage of a *killer* strain for nanoparticle bio-assisted synthesis, and the effectiveness of a conjugated metallic–Kcpf nanoparticle as a novel antimicrobial agent.

**Figure 4:**
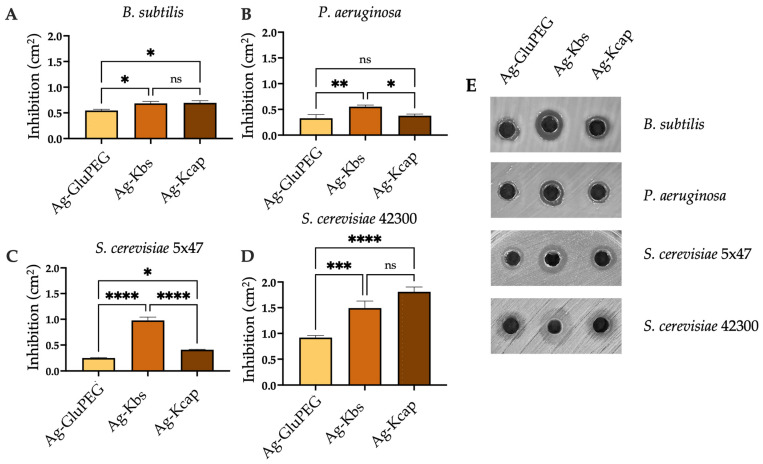
Antimicrobial activity of AgNPs. Inhibition zones of the Ag-GluPEG, Ag-Kbs, and Ag-Kcap against (**A**) *B. subtilis*, (**B**) *P. aeruginosa*, (**C**) a sensitive *S. cerevisiae* 5 × 47, and (**D**) the *killer* strain *S*. *cerevisiae* 42300. Representative images of the inhibition halos are shown in the (**E**) panel. * Significant differences in antimicrobial activity are presented with the *p* values 0.0332 (*), 0.0021 (**), 0.0002 (***), <0.0001 (****).

The results of the inhibition assays reveal an interesting behavior when the Kcpf is on the surface of the NP. The antimicrobial activity of the complex NP-*killer* increases the inhibitory effect of the NP in comparison to the Ag-GluPEG (Figure 4). In this assay, it is compared to the deadly effect of the different preparations of NPs over two bacterial strains, *B*. *subtilis* (Gram+) and *P. aeruginosa* (Gram−), and two yeast strains, *S. cerevisiae* 5 × 47 and *S. cerevisiae* 42300. Interestingly, all NPs demonstrate a strong antibacterial effect. In comparison, the Ag-Kbs and Ag-Kcap NPs had increased inhibitory activity against *B. subtilis* and yeast (*p* > 0.05). However, for the case of *P. aeruginosa,* both Ag-GluPEG and Ag-Kcap nanoparticles showed similar effects, but the Ag-Kbs exhibited a higher effect on the inhibition of this strain.

For the case of yeast, we expected to observe an increase in the inhibition according to the preparation, while those containing Kcpf would have higher activity in the sensitive strain due to the interaction of the *killer* toxin with the cell membrane. It is known that the interactions of the *K*1 *killer* toxin with the receptor β-1-6-D-glucan, as well as the proteins Kre1p [16] and the potassium channel Tok1p [11,12], are key for the susceptibility of the cell to be killed by the *killer* toxin. In our *killer*-containing nanoparticles, we demonstrate an increase in the inhibition of the sensitive strain due to the interaction of the proteins involved in the *killer* system (*p* > 0.01). For the other strain, and contrary to what we expected, the 42300 *killer* strain is more sensitive to the AgNPs, and when the Kcpf is present in the nanocomposite, the activity of this complex is highly increased (*p* < 0.01). Furthermore, the activity of the Ag-GluPEG is lower than that of the NP-K for all the preparations tested. This effect can be related to the mechanism of action and the concentration of *K*1 of each NP. The availability of *K*1 is increased when the NP carries it [56], allowing the toxin to interact more efficiently with the membrane of the cell and increasing the mobilization of the toxin across it and/or to the interior of the cell. Previous studies have explored the basis of the immunity of the *killer* strain to its own toxin, remarking on the importance of the internal blockade of the channel Tok1p [11], and the interaction of the gamma subunit of the *K*1 pptox, when this subunit is removed by Kex1 and Kex2 from *K*1 pptox [10]. However, the immunity of the *killer* system 1 is poorly understood. Our data highlight the enhanced antimicrobial activity of the hybrid metal–protein nanocomposite, suggesting that the presence of Kcpf modifies nanoparticle–cell interactions and increases the susceptibility of *killer* yeast strains to AgNP-mediated inhibition.

### 3.4. Determination of MIC and IC50 Values for AgNPs

**Figure 5:**
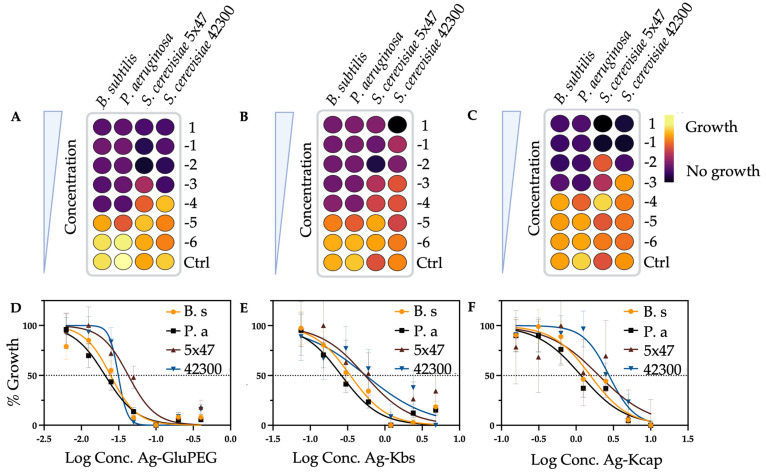
MIC and dose–response modeling to determine the dynamic differences of the NPs. (**A**–**C**) Heatmaps present the normalized absorbance (O.D. at 600 nm) response of the bacterial and yeast strains to serially diluted Ag-GluPEG (**A**), Ag-Kbs (**B**), and (**C**) Ag-Kcap nanoparticles. The MIC reported is the concentration at which there is no visible growth of the microorganism. (**D**–**F**) Dose–response curves of the normalized data are presented in the bottom panels. IC_50_ showed differences in the sensitivity of each microorganism against the different nanoparticles. The heatmap values are represented in arbitrary units. Heatmaps were generated in BioRender.

The development of novel antimicrobial agents involves several approaches, including the evaluation of efficacy and safety of new, improved, or modified antimicrobial agents. To do so, methodologies involving the effect of novel antimicrobial agents on microorganisms can be assessed using standard or modified analyses like disk diffusion, agar dilution, and broth dilution [57]. Here, we combine several methodologies to gain a better understanding of the antimicrobial effect. For the MIC determination, a microdilution on a 96-well plate was established. MIC was defined as the lowest concentration exhibiting no detectable microbial growth [58]. The results of this analysis demonstrate a clear tendency of sensitivity of the bacterial strains, in contrast to yeast. For all three preparations, both *B*. *subtilis* and *P. aeruginosa* exhibit the same MIC: for the case of Ag-GluPEG, the MIC value was 0.025 mg/mL; meanwhile, for Ag-Kbs it was 0.3 mg/mL and 1.25 mg/mL for Ag-Kcap. However, in the case of yeast, we observed marked differences among the sensitive and *killer* strains, with *S. cerevisiae* 42300 more sensitive to all preparations. Overall, the yeast model is less sensitive to the NPs compared to bacteria. The MIC values pre preparation for 5 × 47 and 42300, respectively, are: Ag-GluPEG 0.1 mg/mL and 0.05 mg/mL; Ag-Kbs 1.2 mg/mL for both; and 5 mg/mL and 2.5 mg/mL for Ag-Kcap. Summarizing the data, this indicates a differential activity of the NPs depending on the type of synthesis and the presence of protein-associated components on the nanoparticle surface. Figure 5A–C clearly contrast the differences in MIC in a heatmap, demonstrating that the Ag-GluPEG and Ag-Kbs had the lowest inhibitory concentrations in comparison to the Ag-Kcap. Even though the NPs themselves had different weights, the dilution at which both preparations exhibit the MIC is the same for bacteria (dilution 5). In contrast, the Ag-Kcap has its MIC at dilution 4 for bacteria, dilution 2 for 5 × 47, and dilution 3 for 42300. These data strongly suggest that metal–protein interactions on the synthesis and NP conjugation during stabilization contribute to differences in antimicrobial activity, and they are equally efficient as a chemically synthesized NP.

The relative concentrations of silver and organic matter in the three preparations were not directly quantified in this study. Total nanoparticle concentration is reported as the mass of the washed pellet per unit volume of PBS (mg/mL), consistent with prior protocols for chemically and biologically synthesized AgNPs [59,60,61]. This unit is transparent and reproducible but does not allow direct cross-comparison of silver dose between preparations, since the Kcpf-containing pellets contain a lower mass fraction of Ag^0^ than the Ag-GluPEG pellet. Preliminary EDS analysis confirmed the presence of Ag, C, N, as well as other elements from the organic corona, in agreement with the HR-STEM observations. Future studies using ICP-OES, atomic absorption spectroscopy, or thermogravimetric analysis will be conducted to determine the absolute Ag content of each preparation.

To better understand the differences in toxicity and potency between the AgNPs, a dose–response analysis was conducted. The curves obtained demonstrate the differences in each preparation on the microorganisms. The antimicrobial response was represented as the percentage reduction in microbial growth as a function of nanoparticle concentration, as is demonstrated in Figure 5D–F; the nanoparticles act differently on every microbial system. For all three preparations, the bacterial models were significantly more susceptible, exhibiting lower IC_50_ values compared to yeast models. Notably, *P. aeruginosa* is the most susceptible microorganism for all the preparations, with IC_50_ values of 0.02081, 0.2622, and 1.167 mg/mL for Ag-GluPEG, Ag-Kbs, and Ag-Kcap, respectively. This effect can be driven by the structural composition of the external membrane of Gram-negative bacteria. Due to the presence of a lipopolysaccharide (LPS) network, their negative charge allows the Ag^+^ ions to be attracted more efficiently to the membrane, leading to fast destabilization and growth inhibition of the bacteria [62]. Additionally, the presence of PEG-associated surface properties may influence nanoparticle–membrane interactions and silver ion availability [63]. The Ag-GluPEG preparation was more potent than the Kcpf-associated NPs. This substantial difference in potency can be explained by the dynamics of the nanoparticle corona. The PEG coating may facilitate nanoparticle dispersion and influence silver ion availability. On the other hand, the Ag-Kbs and Ag-Kcap containing protein-associated surface layers may reduce the accessibility of silver ions to microbial cells (Figure 3) [64,65]. In consequence, the concentration needed to reach the 50% inhibition of the strain is more elevated. Interestingly, the case of *B. subtilis* is similar to that of *P. aeruginosa,* with a low IC_50_ of 0.02464 mg/mL (Ag-GluPEG) and 0.3441 mg/mL (Ag-Kbs). However, for the Ag-Kcap preparation, the IC_50_ increases to 1.552 mg/mL, indicating that the nanoparticle core blended with the Kcpf forms a barrier that makes it difficult to penetrate the cell-wall structure.

FTIR analysis confirmed the presence of a stable protein coat for Ag-Kbs and Ag-Kcap. Even though we could not determine a specific proportion of *killer* toxin on the coating, we suggest that *killer* toxin-associated activity contributes to the antimicrobial behavior of the nanocomposite. This synergistic effect explains the shift observed in the *killer* strain compared to the sensitive strain (Figure 5E). Using the Ag-GluPEG baseline, the chemically synthesized NPs inhibit both strains of *S. cerevisiae* through a highly cooperative mechanism (Hill slope of −7.179 for 42300 and −2.390 for 5 × 47), indicating a synchronized threshold effect similar to the “all-or-nothing” effect, where, at a critical particle concentration, the cellular homeostasis may be disrupted. However, when the Kcpf is present in the NP corona, the Hill slope for the *killer* strain is significantly decreased (−1.036), with an IC_50_ of 0.5574 mg/mL. This result can be attributed to a non-cooperative interaction, a reduction in the silver ion release rate, and intrinsic resistance mechanisms associated with the *killer* phenotype. In contrast, the sensitive strain 5 × 47 lacks those resistance mechanisms to the *killer* toxin, whose effect is observed by a cooperative Hill slope of −1.439 and a lower IC_50_ of 0.5418 mg/mL.

An interesting behavior is observed on the Ag-Kcap preparation (Figure 5F). In this assay, the *killer* strain’s Hill slope increases to −2.529 with an IC_50_ of 2.77 mg/mL, whereas the sensitive strain shows a flatter Hill slope of −1.276 and an IC_50_ of 2.026 mg/mL. In this preparation, the mixture of stable PEG-coated NPs and the Kcpf interacts in a loose secondary outer layer. For the case of the strain 5 × 47, the nanocomposite generates a gradual cooperative mechanism of death (Hill slope of −1.276). Meanwhile, for the *killer* strain, the combination of PEG coating and *killer* protein fractions produces a distinct antimicrobial response profile. As shown in Figure 5D, this strain is more sensitive to AgNP toxicity, and in addition to the effect of the Kcpf, the interaction of this preparation results in a steeper concentration-dependent inhibition response (Hill slope of −2.529). Rather than acting as isolated entities, the observed antimicrobial activity stems from a dual synergistic mechanism combining nanoparticle core dynamics and biomolecular surface properties. The metallic silver core serves as a reservoir for toxic Ag^+^ ions that induce membrane permeability and oxidative stress, whereas the outer Kcpf corona supplies active biomolecules capable of specific receptor binding on the cell envelope. This structural synergy effectively dictates the differential cooperativity (reflected in the distinct Hill slope transitions) between sensitive and killer yeast strains.

## 4. Conclusions

The present study evaluates for the first time the synthesis of silver nanoparticles with a *killer* strain of *S*. *cerevisiae*, demonstrating that *killer*-concentrated protein fractions contribute to nanoparticle surface composition and influence their antimicrobial properties. UV–Vis spectroscopy confirmed the characteristic surface plasmon resonance (SPR) band of AgNPs in all formulations. However, there are substantial differences between them. In the first instance, the SPR of the Ag-GluPEG NPs is modulated by the glucose concentration and reaction time. Meanwhile, the incorporation of a Kcpf led to a spectral difference. The biosynthesized and capped NPs showed the SPR of the AgNPs and an intense signal at 280 nm, consistent with the presence of protein-associated components in the nanoparticle preparation.

On the other hand, the physicochemical characterization by HR-STEM, EDS, and FTIR demonstrates clear differences. The Ag-GluPEG exhibit a well-defined cap, while biosynthesized and capped nanoparticles show coatings of proteins and sugars with irregular morphologies, which could be related to the biomolecules present in Kcpf that may contribute to nanoparticle reduction and stabilization. In turn, this corona configuration may influence silver ion availability and nanoparticle–microbial interactions.

We observed a higher susceptibility of bacterial models compared with yeast models. For the case of *P. aeruginosa* and *B. subtilis*, all three preparations exhibited high toxicity compared to the yeast models; this difference may be associated with structural characteristics of bacterial envelopes, including LPS composition in Gram-negative bacteria and cell-wall organization in Gram-positive bacteria. It is interesting to highlight that the Ag-Kbs and Ag-Kcap, despite exhibiting higher IC_50_ values, offered a mechanistic trade-off. Although the Ag-GluPEG had the lowest IC_50_ and thus the highest efficacy, the presence of the *killer* toxin embedded in the protein corona contributed to a broad mechanism of action, combining membrane-associated and oxidative stress to the reported mechanisms of action for *killer* toxin 1.

In the case of the *killer* strain, there was an interesting scenario. While the *killer* strain is immune to its own toxin, the combination of Ag-GluPEG nanoparticles and Kcpf-derived components resulted in increased susceptibility of the *killer* strain to nanoparticle-mediated inhibition. In comparison, when the nanoparticle was biosynthesized, we hypothesize that the Kcpf acts as a dynamic barrier that controls the release of toxic Ag^+^ ions, and the contribution of Kcpf-derived components appears to be limited in the *killer* strain under these conditions. This interaction could be observed by a flattened Hill slope. However, when the Ag-Kcap interacts with the *killer* strain, a synergy of the loose protein layer on a stable PEG-coated AgNP causes a strong and sudden reaction in the yeast, making it very sensitive to the nanocomposite.

These results demonstrate that the incorporation of *killer*-derived proteins into a hybrid nanomaterial is a promising strategy to modulate the antimicrobial activity of silver nanoparticles, while retaining the broad-spectrum activity of the silver core. The data are consistent with a model in which the outer biological corona contributes by binding the nanocomposite to the target cell, while the inner shell controls Ag^+^ release. Nevertheless, three limitations of the present study should be acknowledged: (i) the Kcpf is operationally defined by ultrafiltration and contains co-secreted yeast proteins in addition to *K*1; (ii) the relative proportion of *K*1 within the corona is not yet quantified; and (iii) the antimicrobial assays were performed under short-term culture conditions. Further studies will be conducted to assess (i) long-term colloidal stability and corona preservation, (ii) cytotoxicity against mammalian cell lines, and (iii) activity against bacterial biofilms. Addressing these points will be necessary before any biomedical or food-safety application of these nanomaterials can be considered.

In summary, the bio-assisted synthesis and surface conjugation of silver nanoparticles using killer toxin-containing protein fractions (Kcpf) represent an innovative approach to developing hybrid antimicrobial nanocomposites. While our findings demonstrate promising, strain-dependent antimicrobial efficacy, the present study represents a foundational characterization. Further investigations focusing on long-term colloidal stability, in vitro cytotoxicity against mammalian cell lines, and biological compatibility are essential to fully ascertain the safety and real-world applicability of these nanobiomaterials.

## Data Availability

The data presented in this study are available on request from the corresponding author.

## Supplementary Materials

The following supporting information can be downloaded at: https://www.mdpi.com/article/10.3390/nano16150918/s1, Figure S1: EDS analysis of AgNPs.
