# Supplementary material for: Antimicrobial Effect of Silver Nanoparticles Capped with Killer Yeast-Derived Protein Fractions
Source: Nanomaterials (Basel). 2026 Jul 26;16(15):918. doi: 10.3390/nano16150918 (PMC13467702; doi:10.3390/nano16150918)
Supplement: Supplementary file 1 [file nanomaterials-16-00918-s001.zip › nanomaterials-4469027-supplementary.pdf]

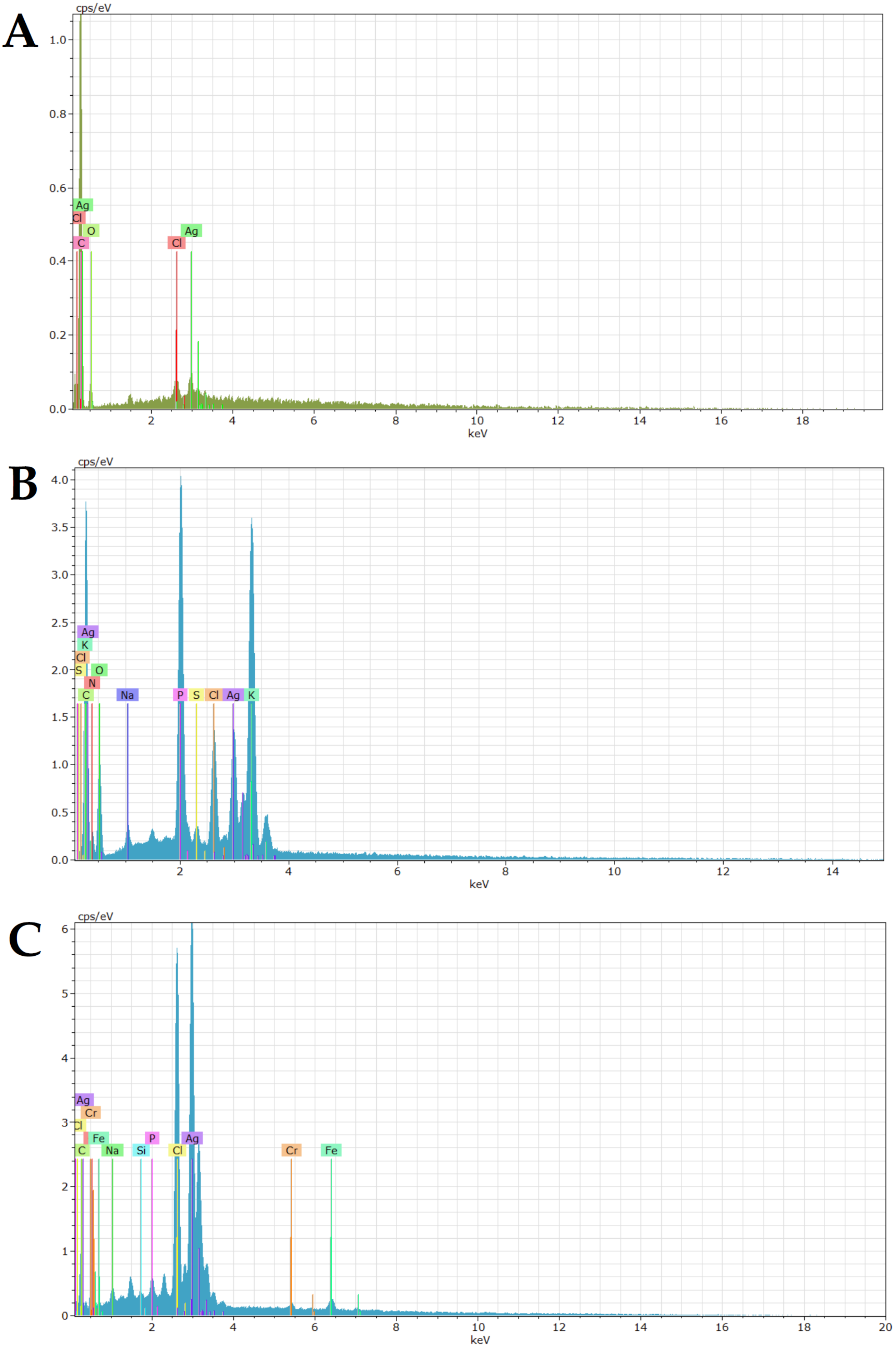

Figure S1. EDS analysis of AgNPs.
